# Is urinary incontinence associated with lichen sclerosus in females? A systematic review and meta‐analysis

**DOI:** 10.1002/ski2.13

**Published:** 2021-02-12

**Authors:** L. Kirby, S. Gran, I. Kreuser‐Genis, C. Owen, R. Simpson

**Affiliations:** ^1^ Department of Dermatology Glasgow Royal Infirmary Glasgow UK; ^2^ Centre of Evidence Based Dermatology School of Medicine University of Nottingham Nottingham UK; ^3^ Department of Dermatology Royal Alexandra Hospital Paisley UK; ^4^ Department of Dermatology East Lancashire Hospitals NHS Trust Blackburn UK

## Abstract

**Background:**

Lichen sclerosus (LS) is a scarring chronic inflammatory disease with a predilection for genital skin in both sexes. The aetiology of LS is controversial, but evidence increasingly suggests that the occluded exposure of susceptible epithelium to urine is involved in the pathogenesis of genital LS in males. This theory has not yet been robustly investigated in females.

**Objectives:**

This review and meta‐analysis examined whether there is an association between urinary incontinence (UI) and genital lichen LS in females.

**Methods:**

We performed a comprehensive search of MEDLINE, Embase and CINAHL to identify observational studies assessing the prevalence of UI in females with LS. DerSimonian and Laird random‐effects models were used to estimate the overall pooled prevalence and risk ratio compared to controls. Heterogeneity was assessed.

**Results:**

In total, eight studies met the inclusion criteria and five studies were included in a meta‐analysis. Three studies were graded as moderate quality and five were poor. The pooled prevalence for UI in LS was 0.35 (95% confidence interval [CI] 0.13–0.58, *I* ^2^ = 98.4%). The risk ratio of UI in LS was 0.97 (95% CI 0.53–1.75, *I* ^2^ = 87.5%).

**Conclusion:**

There appears to be no difference between patients with LS and those without LS in terms of UI. Studies are limited by clinical and methodological quality and heterogeneity is high. Well‐designed prospective studies are needed.

1

**What's already known about this topic?**

- Lichen sclerosus (LS) is a common inflammatory skin condition predominantly affecting the genitalia. It affects both males and females.
- The aetiopathogenesis of LS is controversial.
- Occluded exposure to urine is implicated in the pathogenesis of genital LS in males. It therefore follows that urine may also be a precipitant in females.

**What does this study add?**

- This is the first study to evaluate the evidence available for the prevalence of urinary incontinence (UI) in vulval LS compared to controls.
- From the evidence identified, prevalence of UI in patients with vulval LS is no different to the general population.
- From the evidence identified there is no difference in UI between patients with vulval LS and control groups.
- The existing evidence is limited and quality is poor.

## 2 INTRODUCTION

Lichen sclerosus (LS) is a scarring chronic inflammatory disease that has a predilection for genital skin in both sexes.^1^ The aetiology of LS is likely multifactorial, but an increasing amount of evidence suggests that occluded exposure of susceptible epithelium to urine is involved in the pathogenesis of LS in males.^2^, ^3^, ^4^ In males, droplets of urine can become occluded between the penis and foreskin.^5^ This may explain the pattern of LS seen in males which predominantly involves the distal penis and foreskin. Involvement of the perianal region in men with LS is extremely rare.^6^ In contrast, LS in females typically has a “figure‐of‐eight” distribution involving the labia minora, interlabial sulci and perineum. The vulva is exposed to urine in all women, but these areas are most likely to have occluded exposure to urine when incontinence occurs. LS does not typically affect the glycogenated mucosa of the introitus.^2^ The role of occluded exposure to urine is further supported by the finding that LS is very rare in circumcized males.^7^ Peristomal LS occurs in patients with urostomies but is seen only rarely in those with ileostomies, suggesting that the triggering factor is specifically related to urine and more than simply moisture on the skin.^8^

Identifying potential trigger factors for LS was identified as one of the top 10 questions in the Lichen Sclerosus Priority Setting Partnership; *“Can lichen sclerosus be prevented from occurring and what are the trigger factors?”* ^9^ “Trigger factors” include both factors responsible for development of lichen sclerosus and for its flare‐ups; for example, irritation from clothing, chemicals or urine, trauma, environmental factors, drugs and medications.

To date, studies examining the association between urinary incontinence (UI) and genital LS have been mostly limited to males. Extrapolating established evidence in males to female patients with LS, it is possible that occluded exposure to urine is a trigger factor for LS. If the association is positive, treatment of incontinence would become an essential part of the management of women with LS and in females who might be at increased risk of LS, for example, those with affected family members. Longer term, this could contribute to an evidence base for ways of preventing LS in a susceptible population.

The aim of this systematic review and meta‐analysis was to evaluate whether there is an association between UI and genital LS in females in the existing literature. The population being studied were females (adults and children) with genital LS. For cross‐sectional and cohort studies, the control group were cases without LS. The outcome for these studies was pooled prevalence and risk ratio of UI developing in patients with LS. For case‐control studies, the control groups were cases without UI and the outcome was the odds ratio (OR) for developing UI in the presence of LS. Case‐control studies where the case group had LS and control group did not have LS were also included in the search strategy.

## 3 METHODS

This review was registered with the Prospective International Register for Systematic Reviews (PROSPERO) number CRD42020164396 and reported in line with PRISMA guidelines. The search strategy was devised with the assistance of an information specialist. Electronic databases MEDLINE, Embase and CINAHL were searched in July 2020 from inception. Keywords were “lichen sclerosus et atrophicus” or “lichen sclerosus” or “lichen sclerosis” and “urinary incontinence” or “incontin*”. A full search strategy is available (Appendix S1). Grey literature sources EThOS, DART‐Europe, OpenThesis and Google Scholar were searched at the same time. References of key studies were hand searched and a citation search in Google Scholar was conducted on five key included studies. Articles in languages other than English were translated.

### 3.1 Eligibility

Prospective and retrospective cohort studies, case‐control studies, cross‐sectional studies and case series were included. Studies with less than five cases were excluded, as these would have low precision. All languages were included. Included studies were required to have a clear definition of the study population (adult and paediatric females with genital LS, as diagnosed either clinically or histologically) and documented exposure to UI. Exclusion criteria were studies involving males only, studies focusing on extragenital LS where genital LS was not described and studies involving less than five cases. Studies considered for meta‐analysis were required to report prevalence of UI, with clear separate reporting for females if males were included.

### 3.2 Data extraction

A standardized extraction form was piloted and used to extract data for evidence synthesis. Two review authors (Lisa Kirby and Inge Kreuser‐Genis) extracted the data independently and any discrepancies were identified and resolved through discussion with a third author (Rosalind Simpson). Year of publication, country and type of study were extracted. The total number of study participants and the number of participants included in analysis were recorded. The method of diagnosis of LS (clinical or histological), exclusion of cases of LS/lichen planus (LP) and details of the comparator group were recorded. We considered exclusion of LS/LP overlap cases to be important in order to accurately evaluate the evidence in cases of pure LS. When LS and LP overlap, the condition behaves differently to pure LS, and identifying the predominant condition can be challenging. Data for adults and children were recorded separately. An interpretation summary including outcome, confidence intervals and statistical significance were produced for each study.

### 3.3 Quality assessment

Methodological quality was assessed and recorded using the Critical Skills Appraisal Programme (CASP) tools for case control and cohort studies.^10^ The following criteria were defined prior to extraction and used in addition to CASP: good–use of a validated score for diagnosis of UI, clear exclusion of LS/LP overlap, clear method of LS diagnosis stated; moderate–use of validated score for diagnosis of UI but no information on LS/LP overlap or no information on methods of LS diagnosis; poor—no use of validated score for diagnosis of UI.

### 3.4 Statistical analysis

Meta‐analyses using DerSimonian‐Laird random‐effects model^11^ were carried out for pooled prevalence and relative risk (RR) of UI occurring in patients with LS. Studies that were not included in meta‐analyses (*n* = 3) have been described narratively. Heterogeneity was assessed using *I* ^2^. Subgroup analyses were conducted for study quality and utilization of a validated UI diagnostic tool, to explore heterogeneity. Publication bias could not be assessed as less than 10 studies were included. All statistical analysis was carried out using Stata version 16.

## 4 RESULTS

**FIGURE 1:**
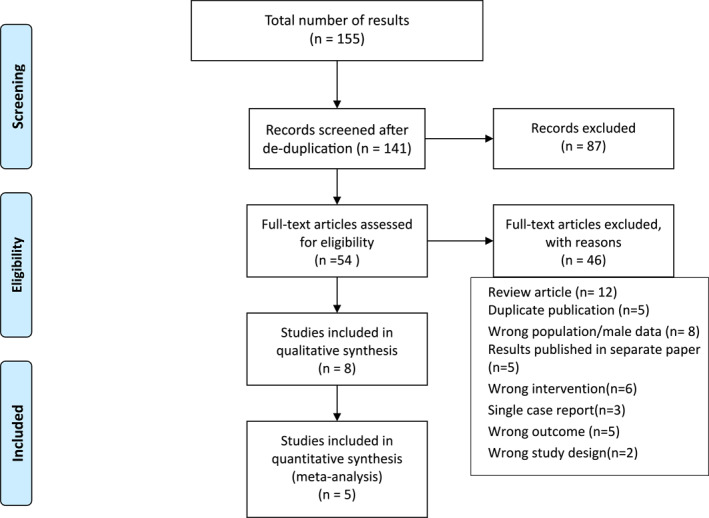
Study flow chart

The search identified 155 studies from electronic databases and 22 from grey literature and secondary search sources. Following deduplication, 141 studies were screened. Fifty‐four articles met our eligibility criteria for full‐text screening. Eight studies were included and 46 were excluded, with reasons recorded (Figure 1).

### 4.1 Study description

Five cross‐sectional studies, two cohort studies and one case series met the inclusion criteria. The total number of participants within the included studies was 3127. The study population included for analysis totalled 1991 adults and 32 children. The participant age range in the included studies was between 3 and 100 years. In the two studies where age was reported separately for case and control groups, age for the LS group was significantly higher compared to controls; median age 58 versus 40, *p* < 0.05,^12^ mean age 57.8 ± 13.7 versus 47.1 ± 15.1, *p* < 0.001 in vulval disease controls and mean age 57.8 ± 13.7 versus 41.5 ± 13.8 , *p* < 0.001 in annual gynaecology exam controls.^13^ Five studies were conducted in Europe and three in the US. The majority were carried out in tertiary centres, in keeping with the typical setting of specialist vulval clinics. For diagnosing LS, one study used histology to diagnose all cases included. The majority of studies (*n* = 5) employed a combination of clinical examination and biopsies to confirm LS, which is consistent with routine clinical practice. No detail was provided regarding the diagnosis of LS in two studies. Exclusion of cases of LS/LP overlap was documented in two studies, with no detail in the rest. Methods of diagnosing UI were also varied. Patients self‐reported UI in one study in a simple “yes/no” questionnaire. Validated questionnaires were used in three studies, with one of these studies also including the use of physical testing for stress UI. No detail was provided about the methods used to diagnosis UI in three studies.

Four studies included controls; routine gynaecology annual examination patients (*n* = 1), gynaecology patients with lower urinary tract symptoms (LUTS; *n* = 1), a combination of patients with non‐lichenoid vulval disease and routine gynaecology annual examination (*n* = 1) and patients with LP (*n* = 1). Two further studies included national prevalence data for UI as a comparator.

In one cross‐sectional study involving four cohorts of patients,^14^ a group was extracted from the overall study population for analysis according to inclusion criteria. Cohorts A and B could not be included because patients were assessed for presence of general LUTS with a single yes/no question, therefore specific detail regarding UI was not available for extraction. Cohorts C and D were included in the meta‐analysis as prevalence of UI was reported.

Authors were contacted for additional data for three studies. One of these studies included males in the overall population; female participant data were provided by correspondence with the authors, allowing this study to be included in the meta‐analysis.^15^ Additional data regarding prevalence of UI in case and control groups could not be provided from the other two studies, which reported OR^12^ and RR^16^ therefore they have been narratively described.

### 4.2 Methodological quality

Three studies were graded as “moderate” quality and five were “poor”. Absence of a validated score for diagnosis of UI was the most common reason for downgrading (*n* = 5). Where a validated score was used but no information was provided on exclusion of LS/LP overlap and/or method of LS diagnosis, studies were also downgraded (*n* = 2). A single study met the criteria for “good”, however was downgraded to “moderate” due to missing data.^13^

### 4.3 Meta‐analyses

Meta‐analyses for pooled prevalence and RR were performed using data from five studies (Table 1). The remaining three studies that were not included in the meta‐analysis have been narratively described (Table 2). The total number of children in the included studies was small (*n* = 32), therefore data for children and adults were combined for pooled prevalence meta‐analysis.

**TABLE 1:** Studies included in meta‐analysis

Studies, country	Type of study	Total study participants (*n*)	Age of participants (mean/median/range)	Participants included in analysis (*n*)	Number of cases(*n*)	Number of controls (*n*)	Diagnosis of LS (clinical/biopsy)	Exclusion LS/LP overlap	Diagnosis of UI	Comparator	Outcome	Comparator group	Statistical significance (*p*)	Quality
Berger et al.^20^ USA	Cohort	308	Median 56.4, range 20.0–92.5	308 Adults 0 children	308	0	Clinical with some biopsies	Yes	Nonvalidated questionnaire	Published prevalence rates in general population	Prevalence UI 38.6% (*n* = 119)	Prevalence UI 33%	0.02	Poor
Christmann‐Schmid et al.^14^ Switzerland	Cross‐sectional	725	Range 20–100	373 Adults 0 children^a^	113	260	Clinical with some biopsies	Not documented	Validated questionnaire (GFPFQ) plus urodynamic studies	Urogynaecology patients with LUTS, without LS/other vulval disease	Prevalence UI 23% (*n* = 26)	Prevalence UI 33.1% (*n* = 86)		Moderate
Ismail et al.^21^ UK	Cohort	26	Range 3–17	0 Adults 26 children	26	0	Clinical with some biopsies	Not documented	Self‐reported history	None	Prevalence UI 38% (*n* = 10)	None		Poor
Swenson et al.^13^ USA	Cross‐sectional	331	LS mean 57.8 ± 13.7, non‐lichenoid vulval disease mean 47.1 ± 15.1, routine annual exam mean 41.5 ± 13.8	284 Adults 0 children	89	195	Biopsies	Yes	Validated questionnaire (MESA)	Non‐lichenoid vulval disease patients and routine gynaecology annual examination patients	Prevalence UI 70.8% (*n* = 63)	Prevalence UI 55% (*n* = 108)		Moderate
Virgili et al.^15^ Italy	Cross‐sectional	729	Range 6–97, mean 57.3 ± 17.4	386 Adults 6 children^b^	392	0	Clinical with some biopsies	Not documented	No detail	Published prevalence rates in general population	Prevalence UI 7.4% (*n* = 29)	Prevalence UI 7.4% (6.7% age adjusted)		Poor

Abbreviations: OR, odds ratio; RR, risk ratio.

^a^ Cohorts C and D analyzed.

^b^ Female only data provided by author.

**TABLE 2:** Studies not included in meta‐analysis

Studies, country	Type of study	Total study participants (*n*)	Age of participants (mean/median/range)	Participants included in analysis (*n*)	Number of cases (*n*)	Number of controls (*n*)	Diagnosis of LS (clinical/biopsy)	Exclusion LS/LP overlap	Diagnosis of UI	Comparator	Outcome	Comparator group outcome	Statistical significance	Quality
Bratila et al.^17^ Romania	Case series	22	Range 52–73	22 Adults 0 children	22	0	Unclear	Not documented	No detail	None	Prevalence UI 90% (*n* = 20)	None		Poor
Kennedy et al.^12^ USA	Cross‐sectional	645	LS group median 58, range 23–81. Annual exam group median 40, range 19–80	277 Adults 0 children	43	234	Clinical with some biopsies	Not documented	Validated questionnaire (interstitial Cystitis Symptom Index and UDI‐6)	Routine gynaecology annual examination patients	OR UI 0.4	None	95% CI (0.2–1.0)	Moderate
Vieira‐Baptista et al.^16^ Portugal	Cross‐sectional	341	No detail	341 Adults 0 children	255	86	Unclear	Not documented	No detail	Patients with LP	RR of UI in LS versus LP 2.40	NA		Poor

### 4.4 Prevalence of UI in LS

**FIGURE 2:**
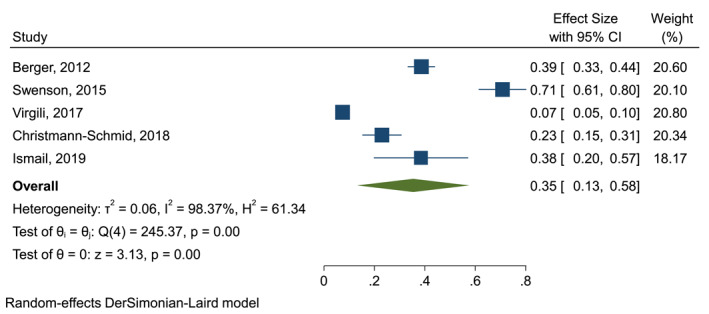
Random‐effects meta‐analysis for pooled prevalence of urinary incontinence in lichen sclerosus

Five studies (1383 patients) were included in a random‐effects meta‐analysis for pooled prevalence (Figure 2). The pooled prevalence for UI in LS was 0.35 (95% CI 0.13–0.58). Prevalence varied considerably between studies (7.4%–70.8%) Heterogeneity was high (*I* ^2^ = 98.4%).

### 4.5 Risk ratio of UI in LS

**FIGURE 3:**
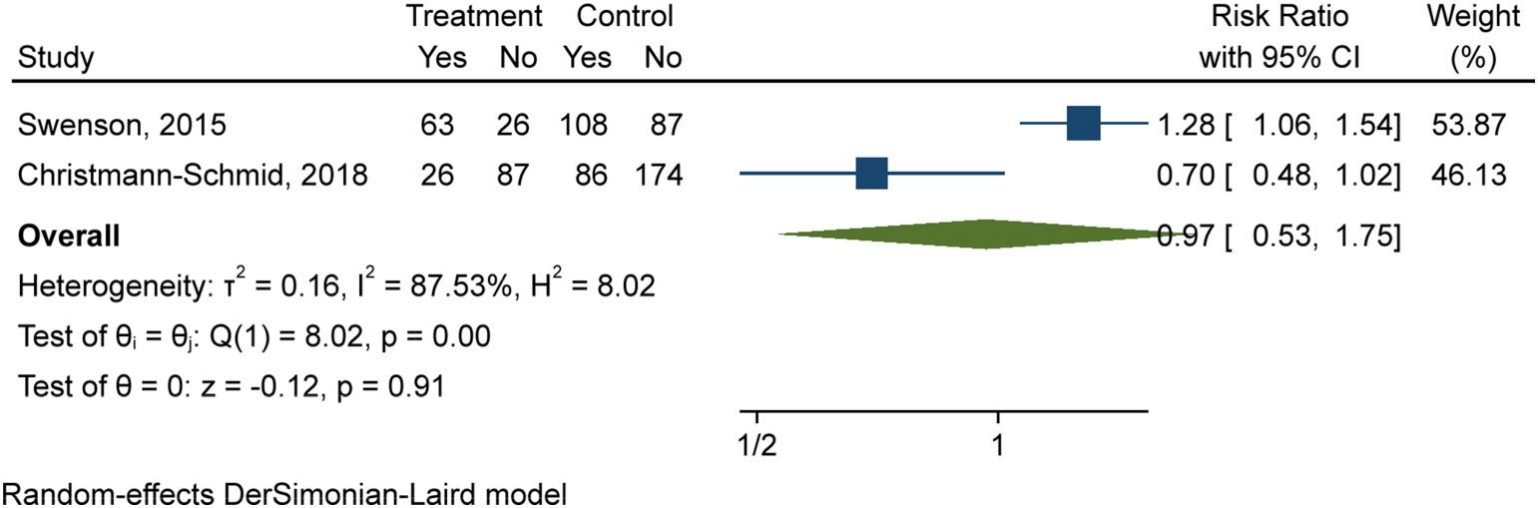
Random‐effects meta‐analysis for risk ratio of urinary incontinence in lichen sclerosus

Two studies included data that allowed calculation of RR (202 cases, 455 controls), shown in Figure 3. The control groups included 260 urogynaecology patients with LUTS but without LS or other vulval disease, 82 patients with non‐lichenoid vulval disease and 113 routine annual gynaecological examination patients. Random‐effects meta‐analysis showed the combined risk‐ratio of UI in LS was 0.97 (95% CI 0.53–1.75). Heterogeneity was high (*I* ^2^ = 87.5%).

### 4.6 Subgroup analysis

Subgroup analysis for prevalence of UI in LS by study quality was conducted (Figure S2). Heterogeneity remained high in the two groups (*I* ^2^ = 98.2% in the poor group, 98.3% in the moderate group). Subgroup analysis for studies which utilized a validated tool for diagnosis of UI yielded the same results, as this was a key criterion for defining quality. We were not able to conduct any other subgroups due to insufficient studies.

### 4.7 Descriptive analysis

Three studies could not be included in meta‐analysis for reasons outlined above. One study was a case series of 22 patients with LS. UI was present in 90% of cases; however, the method of diagnosis of UI was unclear.^17^ A further cross‐sectional study compared rates of UI in several vulval diseases including LS to control patients attending for annual gynaecology examination. The validated Urinary Distress Inventory short form score (UDI‐6) was used to diagnose UI. Comparing 43 patients with LS to 234 controls, the OR for UI in LS was 0.4 (95% CI 0.2–1.0), adjusted for BMI, age, parity and education. Interestingly, UDI‐6 score was statistically higher among all vulval disease diagnoses, compared to controls (*p* < 0.01).^12^ An additional cross‐sectional study compared UI in LS to LP. As mentioned previously, there can be significant overlap between LS and LP, therefore LP is not a good comparator. No detail was given on the method of diagnosis of UI. The RR of UI in LS versus LP was 2.40 (CI not reported).^16^

## 5 DISCUSSION

This review has shown that the pooled prevalence for UI in patients with LS in these studies is 35% (95% CI 13%–58%). We know that UI is common in females and increases with age. Prevalence estimates for UI in the general population vary widely according to populations studied, definitions of UI and methods employed in diagnosis. One large European study reported an overall prevalence of around 35% across countries.^18^ The risk ratio for UI in LS compared to controls in the two studies included was 0.97 (95% CI 0.53–1.75), therefore suggesting there is no difference in risk between the groups. It is not possible to infer a temporal relationship between LS and UI based on the findings of this review; however, this is the first review summarising data on the prevalence of UI in LS.

The studies included in this review have many limitations. The majority of the studies were cross sectional, therefore exposure and outcome were measured at the same time. The direction of the association cannot be established. Many of the studies are small and potentially underpowered. There was lack of adjustment for confounding factors in several studies. In terms of population, all studies were performed in developed countries and many stated that the study population was entirely Caucasian. Where sufficient detail was given, the majority of patients were identified from specialist vulval clinics; potentially a population with more severe disease which may not be representative of the wider population of LS patients. All studies were from single centres except Virgili et al.,^15^ which provided data from 15 centres across Italy.

Although it is standard practice for epidemiological studies investigating UI to utilise self‐reporting questionnaires, this is an inherent source of bias. Patients selected from specialist vulval clinics may be more motivated to return questionnaires, particularly if asked to complete while attending clinic. UI may be underreported due to embarrassment, or equally overreported in anticipation of treatment gain. There was widespread absence of validated methods for diagnosing UI, further compounded by incomplete or absent reporting on methods used.

Lack of detail regarding the exclusion of LS/LP overlap cases is another common limitation encountered in these studies. Although the majority of cases of LS are diagnosed clinically in routine practice, it is important to confirm the diagnosis histologically in cases which are not entirely typical of LS. Cases of uncertain diagnosis or features of both LS/LP, which can coexist and are difficult to tell apart histologically,^19^ should be excluded to allow for accurate analysis of UI in true LS cases.

Use of valid control groups was variable. Controls were selected from patients attending routine annual gynaecological examination, urogynaecology patients with known LUTS, patients with LP and patients with non‐lichenoid vulval disease. Arguably the only valid control groups were routine annual gynaecology examination patients and non‐lichenoid vulval disease patients.

At review level, there were also limitations. The high levels of heterogeneity mean that the results must be interpreted with caution. We were unable to explore heterogeneity beyond subgroup analysis for quality and use of validated method of diagnosing UI. Publication bias could not be assessed.

This is the first systematic review examining the prevalence of UI in females with LS. Although it has shown that the prevalence of UI in LS is comparable to the general population, caution must be exercized in interpreting these results due to the limited quality and small number of included studies. Good quality prospective studies examining the prevalence of UI in patients with LS compared with controls are needed. Adjustment for confounding factors like age, BMI, history of autoimmunity and family history would allow assessment of how much these variables affect the relationship between UI and LS. Validity could be improved by utilising validated questionnaires for diagnosis of UI as well as exclusion of LS/LP overlap cases. Use of routinely collected electronic healthcare data in a case‐control study would be valuable to examine the relationship at population level.

## CONFLICTS OF INTERESTS

The authors declare that there are no conflict of interests.

## Supporting information

Supplementary Material

Supplementary Material

## References

[1] Lewis F . Dermatoses of the female genitalia: inflammatory dermatoses of the vulva: lichen sclerosus. In: Griffiths CEMBJ , Bleiker T , Chalmers R , Creamer D , editors. Rook's textbook of dermatology. 3. 9th ed. Chicester: John Wiley & Sons; 2016. p. 112.6–9.

[2] Bunker CB , Patel N , Shim TN . Urinary voiding symptomatology (micro‐incontinence) in male genital lichen sclerosus. Acta Derm Venereol. 2013;93(2):246–8. 23093304 10.2340/00015555-1481

[3] Gupta S , Malhotra AK , Ajith C . Lichen sclerosus: role of occlusion of the genital skin in the pathogenesis. Indian J Dermatol Venereol Leprol. 2010;76(1):56–8. 20061733 10.4103/0378-6323.58681

[4] Edmonds EV , Hunt S , Hawkins D , Dinneen M , Francis N , Bunker CB . Clinical parameters in male genital lichen sclerosus: a case series of 329 patients. J Eur Acad Dermatol Venereol. 2012;26(6):730–7. 21707769 10.1111/j.1468-3083.2011.04155.x

[5] Bunker CB . Re: Sanjay Kulkarni, Guido Barbagli, Deepak Kirpekar, et al. Lichen sclerosus of the male genitalia and urethra: surgical options and results in a multicenter international experience with 215 patients. Eur Urol. 2010;58(6):e55‐6. 20864253 10.1016/j.eururo.2010.09.005

[6] Bunker CB , Shim TN . Male genital lichen sclerosus. Indian J Dermatol. 2015;60(2):111–7. 25814697 10.4103/0019-5154.152501 PMC4372901

[7] Mallon E , Hawkins D , Dinneen M , Francics N , Fearfield L , Newson R , et al. Circumcision and genital dermatoses. Arch Dermatol. 2000;136(3):350–4. 10724196 10.1001/archderm.136.3.350

[8] Al‐Niaimi F , Lyon C . Peristomal lichen sclerosus: the role of occlusion and urine exposure? Br J Dermatol. 2013;168 (3):643–6. 22913573 10.1111/bjd.12014

[9] Simpson RC , Cooper SM , Kirtschig G , Larsen S , Lawton S , McPhee M , et al. Future research priorities for lichen sclerosus–results of a James Lind Alliance Priority Setting Partnership. Br J Dermatol. 2019;180(5):1236–7. 30472735 10.1111/bjd.17447 PMC6850137

[10] Critical Skills Appraisal Programme . CASP (case control and cohort studies) checklists. 2018. https://casp-uk.net/casp-tools-checklists/. Accessed 21 November 2020.

[11] DerSimonian R , Laird N . Meta‐analysis in clinical trials. Control Clin Trials. 1986;7 (3):177–88. 3802833 10.1016/0197-2456(86)90046-2

[12] Kennedy CM , Nygaard IE , Bradley CS , Galask RP . Bladder and bowel symptoms among women with vulvar disease: are they universal? J Reprod Med. 2007;52(12):1073–8. 18210896

[13] Swenson CW , Menees SB , Haefner HK , Berger MB . Lower urinary tract and functional bowel symptoms in women with vulvar diseases and controls. Female Pelvic Med Reconstr Surg. 2015;21(4):211–4. 26052645 10.1097/SPV.0000000000000184 PMC4665618

[14] Christmann‐Schmid C , Hediger M , Groger S , Krebs J , Gunthert AR , In cooperation with the Verein Lichen sclerosus . Vulvar lichen sclerosus in women is associated with lower urinary tract symptoms. Int Urogynecol J. 2018;29(2):217–21. 28593367 10.1007/s00192-017-3358-8

[15] Virgili A , Borghi A , Cazzaniga S , Di Landro A , Naldi L , Minghetti S , et al. New insights into potential risk factors and associations in genital lichen sclerosus: data from a multicentre Italian study on 729 consecutive cases. J Eur Acad Dermatol Venereol. 2017;31(4):699–704. 27515901 10.1111/jdv.13867

[16] Vieira‐Baptista P , Cavaco‐Gomes J , Lima‐Silva J , Machado L , Amaral A , Beires J , et al. On the interference of lichen sclerosus and lichen planus with sexual function and how to minimize it. J Low Genit Tract Dis. 2013;17(6):S113–4.

[17] Bratila E , Cirstoiu M , Mehedintu C , Berceanu C , Bohiltea R , Toader O , et al. The effect of chronic vulvar dystrophy on urinary continence in patients at climacterium. Maturitas. 2017;100:169.

[18] Hunskaar S , Lose G , Sykes D , Voss S . The prevalence of urinary incontinence in women in four European countries. BJU Int. 2004;93(3):324–30. 14764130 10.1111/j.1464-410x.2003.04609.x

[19] Day T , Moore S , Bohl TG , Scurry J . Comorbid vulvar lichen planus and lichen sclerosus. J Low Genit Tract Dis. 2017;21 (3):204–8. 28369011 10.1097/LGT.0000000000000307

[20] Berger MB , Damico NJ , Menees SB , Fenner DE , Haefner HK . Rates of self‐reported urinary, gastrointestinal, and pain comorbidities in women with vulvar lichen sclerosus. J Low Genit Tract Dis. 2012;16(3):285–9. 22622339 10.1097/LGT.0b013e3182562f1e PMC3404184

[21] Ismail D , Owen CM . Paediatric vulval lichen sclerosus: a retrospective study. Clin Exp Dermatol. 2019;44(7):753–8. 30623460 10.1111/ced.13894

