# Supplementary material for: Is urinary incontinence associated with lichen sclerosus in females? A systematic review and meta‐analysis
Source: Skin Health Dis. 2021 Feb 12;1(1):e13. doi: 10.1002/ski2.13 (PMC9060132; doi:10.1002/ski2.13)
Supplement: Supplementary file 1 — Supplementary Material [file SKI2-1-e13-s001.pdf]

Database: Ovid MEDLINE

1 Lichen Sclerosus et Atrophicus/
2 ("lichen sclerosis" or "lichen sclerosus").mp.
3 1 or 2
4 exp Urinary Incontinence/
5 incontinen*.mp.
6 4 or 5
7 3 and 6

Database: Embase

1 lichen sclerosus et atrophicus/
2 ("lichen sclerosis" or "lichen sclerosus").mp.
3 1 or 2
4 exp urine incontinence/
5 incontinen*.mp.
6 4 or 5
7 3 and 6

Database: Ebsco CINAHL

1 (MH "Lichen Sclerosus et Atrophicus")
2 "lichen scleros*"
3 1 or 2
4 (MH "Urinary Incontinence+")
5 "incontinen*"
6 4 or 5
7 3 and 6
