# Supplementary material for: Is urinary incontinence associated with lichen sclerosus in females? A systematic review and meta‐analysis
Source: Skin Health Dis. 2021 Feb 12;1(1):e13. doi: 10.1002/ski2.13 (PMC9060132; doi:10.1002/ski2.13)

S2 Subgroup analysis by study quality

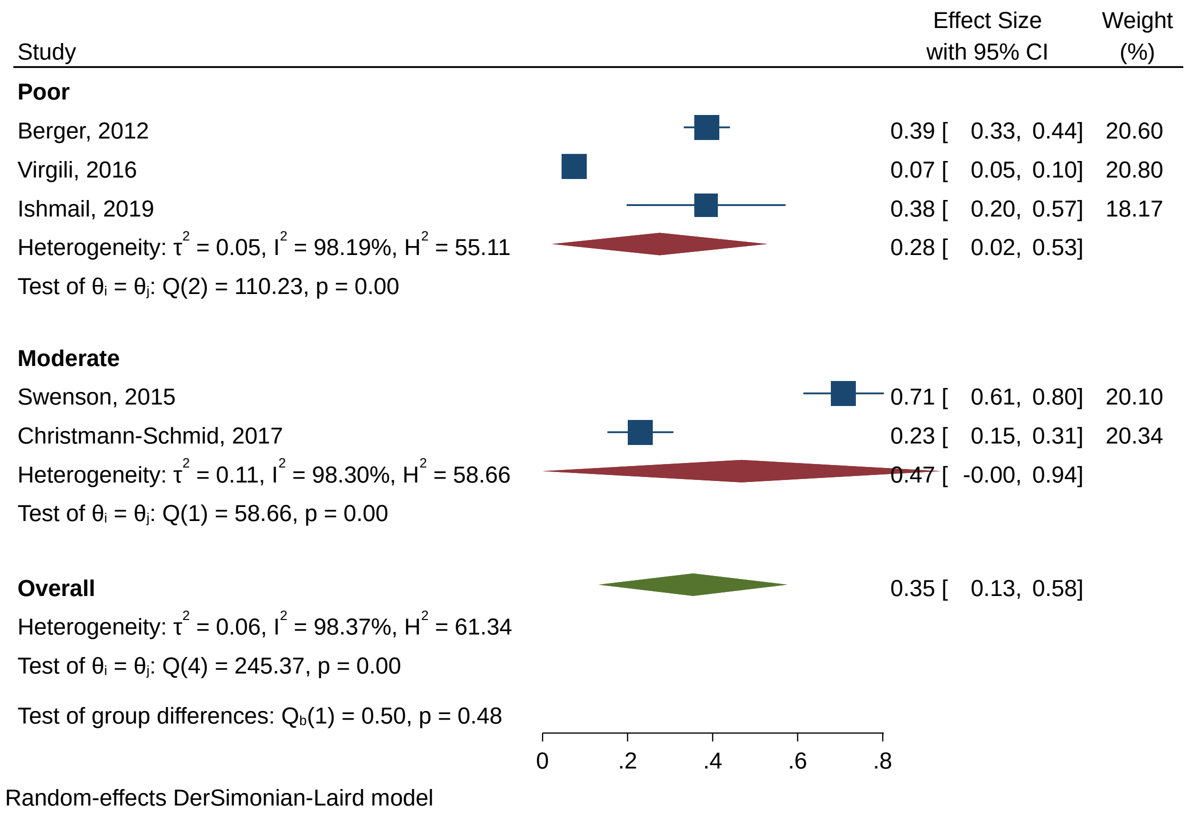

Supplement: Supplementary file 2 [file SKI2-1-e13-s002.docx]
